# A novel micelleplex for tumour-targeted delivery of CRISPR-Cas9 against KRAS-mutated lung cancer†

**DOI:** 10.1039/d4nr03471f

**Published:** 2025-01-13

**Authors:** Siyu Chen, Mariem Triki, Simone Pinto Carneiro, Olivia Monika Merkel

**Affiliations:** a Ludwig-Maximilians-University, Department of Pharmacy, Pharmaceutical Technology and Biopharmaceutics Butenandtstraße 5-13 Munich 81377 Germany siyu.chen@cup.uni-muenchen.de olivia.merkel@cup.uni-muenchen.de

## Abstract

CRISPR-Cas9 has emerged as a highly effective and customizable genome editing tool, holding significant promise for the treatment of KRAS mutations in lung cancer. In this study, we introduce a novel micelleplex, named C14-PEI, designed to co-deliver Cas9 mRNA and sgRNA efficiently to excise the mutated KRAS allele in lung cancer cells. C14-PEI is synthesised from 1,2-epoxytetradecane and branched PEI 600 Da *via* a ring-opening reaction. The resulting C14-PEI has a critical micelle concentration (CMC) of approximately 20.86 ± 0.15 mg L^−1^, indicating its ability to form stable micelles at low concentrations. C14-PEI efficiently encapsulates mRNA into micelleplexes through electrostatic interactions. When the mass ratio is 8 (w/w 8), the C14-PEI formulation exhibits conducive properties, which showed encapsulation efficiency of eGFP mRNA at 99% and led to a 130-fold increase in eGFP expression in A549 cells compared to untreated cells, demonstrating the robust delivery and expression capability of the micelleplexes. Importantly, toxicity tests using intracellular reduction of a tetrazolium salt revealed no significant cytotoxicity, underscoring the biocompatibility of C14-PEI. C14-PEI also shows high efficiency in co-encapsulating Cas9 mRNA and sgRNA, as confirmed by agarose gel electrophoresis. At an sgRNA to Cas9 mRNA molar ratio of 10, the micelleplexes successfully mediate the cutting of mutated KRAS with an indel efficiency exceeding 60%, as determined by the T7 Endonuclease I (T7EI) assay. Droplet digital polymerase chain reaction (ddPCR) further demonstrates that the gene editing efficiency, measured by edited gene copies, is 48.5% in the w/w 4 group and 37.8% in the w/w 8 group. Treatment with C14-PEI micelleplexes containing Cas9 mRNA and sgRNA targeting the KRAS G12S mutation significantly impairs the migration capability of A549 cells and increases apoptosis rates. These findings suggest that C14-PEI effectively disrupts KRAS signalling pathways, leading to reduced tumor cell proliferation and enhanced cell death.

## Abstract 2

CRISPR-Cas9 has emerged as a highly effective and customizable genome editing tool, holding significant promise for the treatment of KRAS mutations in lung cancer.

## Graphical abstract

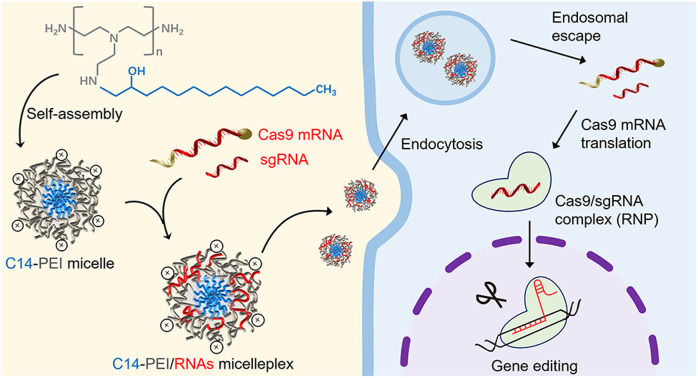

## Introduction

According to the Global Cancer Statistics 2022, lung cancer is the most commonly diagnosed cancer and the leading cause of cancer-related death.^1^ Lung cancer may be induced by a variety of genomic variations, such as *EGFR*, *ALK*, and *MET*. Among these mutations, *Kirsten rat sarcoma 2 viral oncogene homolog* (*KRAS*) mutations are observed in 25% of all cases, making it the most commonly mutated gene.^2^ Although *RAS* genes were the first human oncogenes to be identified, mutant *KRAS* has long been considered an undruggable target due to its spherical structure. The relatively smooth shape of the protein made it difficult to design inhibitors that could bind to surface grooves, stalling progress in drug development for many years.^3^ Despite decades of research, significant progress in *KRAS* drug discovery remained elusive until the pivotal discovery in 2013 of covalently targeting the *KRAS* p.Gly12Cys (G12C) mutation, which catalysed transformative advancements in KRAS-targeted therapy.^4^ To date, two small molecule inhibitors, Sotorasib and Adagrasib, have received accelerated approval for the treatment of non-small cell lung cancer (NSCLC) harbouring *KRAS* G12C mutations.^4^ Moreover, pan-RAS/KRAS inhibitors, combination strategies, and immunotherapeutic approaches have shown significant progress from bench to bedside.^3^ However, the adaptive resistance and toxicity of pan-RAS inhibitors remain challenging drawbacks.

Recently, thanks to the discovery of CRISPR-Cas9, gene therapy has seen exciting developments. Clustered regularly interspaced short palindromic repeats (CRISPRs) are a type of repeat sequences found in prokaryotic bacteria and archaea, functioning as part of their adaptive immune system. CRISPR-Cas9 proves to be an efficient and customizable genome editing tool due to its benefits, such as quick onset, transient expression, low off-target effects (OTEs), and low costs.^5^ It is a promising strategy for regulating gene expression, especially for correcting pathogenic mutations, and could specifically correct *KRAS* mutations.^6^ To deliver CRISPR-Cas9, the most common forms include plasmid DNA,^7^ mRNA/sgRNA,^8^ and protein/sgRNA ribonucleoprotein complexes (RNPs).^9^ Various advantages of the Cas9 mRNA formulation delivery strategy have been reported. First, compared with plasmids, mRNAs only need to enter the cytoplasm to exert its effects.^10^ The delivery of the Cas9 mRNA formulation combined with sgRNA into target cells can express the Cas9 protein transiently, which shortens the duration of gene editing and reduces the chance of OTEs.^5^ Secondly, the intracellular presence of the Cas9 protein is more persistent after mRNA expression compared to the delivery of Cas9-RNPs,^11^ and systemic RNP delivery in clinical settings still requires evaluation compared to more established mRNA-based approaches.^12,13^ During the COVID-19 pandemic caused by the SARS-CoV-2 virus, the rapid development and clinical maturation of mRNA-based vaccines promoted advancements in mRNA delivery techniques.^14^ Additionally, the ability to modify mRNA sequences to encode regulatory elements provides a means to control the expression of gene-editing tools in a cell-specific manner.^10^ In 2013, Shen and colleagues first used the CRISPR-Cas system to cut DNA in zebrafish and mouse embryos using Cas9 mRNA and sgRNA, paving the way for its use in the generation of gene-disrupted animals.^15^

However, unlike mRNA vaccines, the efficiency of each component must be considered in the co-delivery of Cas9 mRNA and sgRNA. Yin and colleagues used nanoparticle-mediated delivery of Cas9 mRNA in combination with adeno-associated viruses (AAVs) encoding an sgRNA and a repair template to edit the *fumarylacetoacetate hydrolase* (*Fah*) gene with significant correction (more than 6%), demonstrating that this method relies on viral co-delivery to supplement mRNA delivery.^16^ Over the past several decades, polymeric nanoparticles have been extensively used to deliver various types of nucleic acids, including plasmid DNA,^17,18^ RNAs,^19–21^ and oligonucleotides,^22,23^ due to their advantages such as facile synthesis, flexible structures and components, ease of functionalization, and degradability.^24^ Polymeric nanoparticles are a collective term used for any type of polymer nano-sized particles, specifically polymer nanospheres and nanocapsules, generally ranging from 100 to 500 nm in size.^25^ Polycationic polymers mediate the encapsulation of CRISPR-Cas9 cargoes into positively charged complexes to enable endocytosis into cells. To date, various polymers have been employed for intracellular CRISPR delivery, such as dendrimers,^26^ Polyethylene glycol (PEG)-based nanocarriers,^27^ poly(β-amino ester)s (PBAEs),^26^ supramolecular polymers,^28^ and degradable polymers (such as polypeptides^9^ and polysaccharides^29^). Polyethylenimine (PEI)-based nanoparticles have demonstrated higher editing efficacy compared to unmodified Cas9/sgRNA complexes with conventional lipids.^30^ Yue and colleagues constructed a graphene oxide (GO)-PEG-PEI nanocarrier for the delivery of high-molecular-weight Cas9/sgRNA complexes, showing that the nanocarrier could be successfully used for efficient gene editing in a human gastric adenocarcinoma cell line (AGS cells) with an efficiency of approximately 39%, while also exhibiting high stability to protect sgRNA from enzymatic degradation.^31^

In this study, following a series of screenings, we designed a novel PEI-based micelleplex, C14-PEI, to deliver Cas9 mRNA and sgRNA targeting mutated KRAS. We tested the encapsulation efficiency and stability of the polymers, characterised the nanoparticles, and evaluated cytotoxicity and eGFP-mRNA expression of the micelleplexes *in vitro* using A549, H1299, and Hop62 cells. The endosomal entrapment and escape of the micelleplex were also investigated using confocal laser scanning microscopy (CLSM). To achieve therapeutically relevant gene editing, Cas9 mRNA and sgRNA targeting mutant *KRAS* were delivered to A549 cells using the C14-PEI micelleplex, and gene editing efficiency was estimated by T7EI assay, ddPCR, and Sanger sequencing. Western blot analysis, cell migration assays, and cell apoptosis assays were conducted to evaluate cellular responses after treatment.

## Experimental

### Materials

1,2-Epoxytetradecane, branched PEI 600 Dalton, 4-(2-hydroxyethyl)-1-piperazineethanesul-fonic acid (HEPES), Dulbecco's Phosphate Buffered Saline (PBS), 0.05% trypsin-EDTA, Tris-buffered saline, Tween 20, RPMI-1640, foetal bovine serum (FBS), Penicillin–Streptomycin solution, 6-diamidino-2-phenylindole dihydrochloride (DAPI), skim milk, heparin, pyrene, paraformaldehyde (PFA), agarose powder, and Cell Counting Kit-8 were purchased from Sigma-Aldrich, Germany. SYBR™ Gold Stain, SYBR Safe DNA Gel Stain, Lipofectamine™ 2000, LysoTracker™ Green DND-26, Annexin V-AF488, GeneArt™ Genomic Cleavage Detection Kit, Phusion Hot Start II High-Fidelity PCR Mastermix, ExoSAP-IT™ Express PCR Product Cleanup Reagent, Pierce™ BCA Protein Assay kit, Novex™ WedgeWell™ 8–16% Tris-Glycin gel, Pierce™ Protease Inhibitor Tablets, RIPA buffer, SuperSignal™ West Femto Maximum Sensitivity Substrat were bought from Thermo Fisher Scientific, Germany. ddPCR NHEJ Gene Edit Assay (primers and probes), ddPCR Supermix for Probes (no dUTP), cartridges, gaskets, droplet generation oil, and droplet reader oil were obtained from Bio-Rad, US. eGFP mRNA (RiboPro, The Netherlands), CleanCap® Cas9 mRNA (5moU) (Trilink Biotechnologies, US), cOmplete™ EDTA-free Protease Inhibitor Cocktail (Roche, Germany), Rotiphorese®NF 10× TBE Buffer (Carl Roth, Germany), propidium iodide (PI) (BD Biosciences, US), DNeasy Blood & Tissue Kit (Qiagen, US), and Amersham™ Protran® western blotting nitrocellulose membranes (Cytiva technologies, Germany) were purchased from the suppliers indicated. Methanol, ethanol, and acetone were provided by Ludwig-Maximilians-University Munich. The primary antibodies for p44/42 MAPK (Erk1/2), Phospho-p44/42 MAPK (T202/Y204), and AKT were from Cell Signaling, US. KRAS polyclonal antibody, Histone-H3 polyclonal antibody, and HRP-conjugated affinipure goat anti-rabbit IgG (H + L) secondary antibody are from Proteintech, Germany. Cy5-mRNA was synthesised and labelled in the laboratory. sgRNA (*KRAS* G12S: 5′-CUUGUGGUAGUUGGAGCUAG-3′) was synthesised by Sigma-Aldrich. Primers for PCR (F: TTTGAGAGCCTTTAGCCGC, R: TCTACCCTCTCACGAAACTC) and primers for Sanger sequencing (F: TCTTAAGCGTCGATGGAG, R: ACAGAGAGTGAACATCATGG) were synthesised by Sigma-Aldrich.

### Cell culture

A549, H1299, and Hop-62 cells were cultured in complete RPMI-1640 medium supplemented with 10% heat-inactivated foetal bovine serum (FBS) and 1% penicillin–streptomycin. All cells were subcultured, maintained, and grown in an incubator at 37 °C in humidified air with 5% CO_2_.

### C14-PEI synthesis and characterization

C14-PEI is prepared by reacting 1,2-epoxytetradecane with branched PEI 600 Dalton (bPEI 600 Da) through a ring opening reaction. Briefly, 1,2-epoxytetradecane and bPEI 600 Da were heated at 95 °C in absolute ethanol for 72 h while stirring. The product was then dialyzed with a 1000 Da cut-off in absolute ethanol, followed by ethanol removal using high-pressure nitrogen gas.^32,33^ The final polymer was confirmed by nuclear magnetic resonance spectroscopy (^1^H NMR) and gel permeation chromatography (GPC).

### Critical micelle concentration

The CMC of C14-PEI was determined using a fluorescence spectrometer with pyrene as the fluorescence probe.^34^ The fluorescence scanning ranged from 300 to 350 nm, and the emission wavelength was set at 373 nm. Pyrene was first dissolved in 0.5 mL of acetone, and the acetone was then allowed to volatilise overnight at room temperature in the dark. The initial polymer solution obtained was diluted into a series of concentrations ranging from 0.0001 to 0.1 mg mL^−1^ and added to vials containing pyrene. The mixture was left to equilibrate in the dark for 24 h before measurement. The final concentration of pyrene in the aqueous solution was 6.5 × 10^−7^ M.

### Micelleplex preparation

Micelleplexes were prepared using C14-PEI and RNA through electrostatic interactions. Briefly, 500 ng of eGFP mRNA and a specific amount of C14-PEI based on mass ratios were dissolved in high-purity water and mixed by pipetting and vortexing in 100 μL of 10 mM HEPES buffer, pH 7.4 or pH 5.4. The mixture was then incubated at room temperature for 1 h. For the co-encapsulation of Cas9 mRNA and sgRNA, a similar method was employed, but Cas9 mRNA and sgRNA were premixed at a molar ratio of 1 : 10 before diluting in the HEPES buffer. The low molecular weight PEI (LMW-PEI, 600 Da) nanoparticles were prepared with the same method. The morphology of the micelleplexes was examined using cryo-electron microscopy (Cryo-EM).

### Micelleplex characterization

The micelleplexes were characterised using a Zetasizer Ultra (Malvern, UK). The micelleplexes suspension was added to a disposable micro-cuvette, and the hydrodynamic diameter and polydispersity indices (PDI) were measured three times per sample using dynamic light scattering (DLS) at a 173° backscatter angle. Subsequently, the same suspension was transferred to a folded capillary cell for each sample to determine the zeta (*ζ*) potential in triplicate using laser Doppler anemometry (LDA), with each run consisting of up to 100 scans. Results are presented as mean ± standard deviation (SD, *n* = 3).

### Encapsulation efficiency test

To evaluate the mRNA encapsulation capacity of C14-PEI, SYBR Gold assays were conducted. SYBR Gold is a cyanine dye that binds to nucleic acids and exhibits fluorescence upon intercalation. Briefly, C14-PEI micelleplexes were prepared as described earlier at weight-to-weight (w/w) ratios of 0, 0.5, 1, 2, 4, 8, and 15, and LMW-PEI nanoparticles were prepared as controls. Subsequently, 100 μL of each micelleplex suspension was added to black FluoroNunc 96-well plates (Fisher Scientific, Germany). A 4× SYBR Gold aqueous solution (30 μL per well) was then added to each well and incubated for 10 minutes in the dark. The fluorescence intensity was measured using a fluorescence plate reader (TECAN, Switzerland) with excitation at 485/20 nm and emission at 535/20 nm.^35^ The fluorescence intensity of free mRNA (w/w = 0) was used as a control and set as 100% fluorescence.
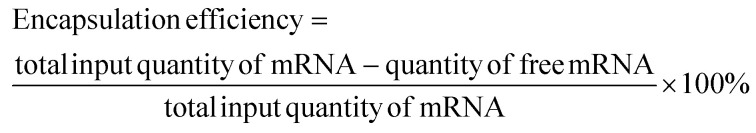


### Heparin competition

To assess the stability of micelleplexes, SYBR Gold assays were conducted in the presence of competing heparin.^36^ Micelleplexes were prepared at a w/w ratio of 8. LMW-PEI nanoparticles at w/w 1 and w/w 8 were prepared as controls. Subsequently, 60 μL of the micelleplex suspension was pipetted into black FluoroNunc 96-well plates (Fisher Scientific, Germany). Next, 10 μL of heparin solutions prepared beforehand at various mass ratios of heparin to mRNA (w/w ratios of 0, 0.5, 1, 2, 4, 6, 8, 10, and 20) were added to each well. After incubating for 30 minutes at room temperature, 30 μL of a 4× SYBR Gold solution was added to each well, and the plate was further incubated for 10 minutes in the dark to allow binding. Fluorescence intensity was measured using a fluorescence plate reader (TECAN, Switzerland) with excitation at 485/20 nm and emission at 535/20 nm. The percentage of free mRNA was calculated by comparing the fluorescence intensity of each sample to that of free mRNA performed as described in section 2.7. All measurements were performed in triplicate, and the results are presented as mean values (*n* = 3).

### CCK-8 cytotoxicity test

The cytotoxicity of micelleplexes was assessed using a cell counting kit-8 (CCK-8) assay in A549, H1299, and Hop-62 cell lines. Specifically, 10 000 cells per well were seeded 24 h prior in a transparent 96-well plate (Fisher Scientific, Hampton, NH, USA). Micelleplexes were freshly prepared at w/w 15, w/w 8, w/w 4 at pH 7.4, and w/w 4 at pH 5.4. After removing the old medium, 100 μL of micelleplex containing medium was added to each well and incubated for 24 h at 37 °C and 5% CO_2_. Subsequently, the medium was aspirated, and fresh medium containing CCK-8 solution (10 μL CCK-8 in 100 μL RPMI-1640 media) was added to each well. After incubating for 3 h, a water-soluble orange formazan product formed in the medium, and absorbance was measured at 450 nm using a Tecan plate reader. The experiment was conducted in triplicate, and the results are presented as mean values (*n* = 3), normalised to the percentage of viable cells relative to untreated cells (100% viability).

### Endosomal escape test by confocal laser scanning microscopy

To visualize the endosomal entrapment behaviour of micelleplexes, A549 cells were imaged using confocal laser scanning microscopy (CLSM, Leica SP8 inverted, software: LAS X, Leica Microsystems GmbH, Germany) after transfection with fluorescent mRNA. Specifically, 10 000 A549 cells were seeded in ibiTreat μ-Slide 8 well plates (ibidi, Germany) and transfected with C14-PEI micelleplexes containing Cy5-mRNA at w/w of 4 and 8. PEI and free Cy5-mRNA served as controls. Following incubation at 37 °C with 5% CO_2_ for 4, 8, or 24 h, cells were stained with LysoTracker Green DND-26 in pre-warmed cell culture medium for 1 h in the cell incubator. After removing the medium, cells were fixed with 4% PFA for 15 minutes in the dark and washed. DAPI was added to appropriate wells at a final concentration of 1 μg mL^−1^ in PBS and incubated for 20 minutes at room temperature in the dark. Subsequently, all cells were washed and maintained in PBS at 4 °C for subsequent analysis using CLSM. Excitation was achieved using a diode laser at 405 nm, an argon laser at 488 nm, and a helium–neon laser at 650 nm. Emission was recorded in the blue channel (420 nm–480 nm) for DAPI, the green channel (500 nm–550 nm) for LysoTracker Green, and the red channel (650 nm–720 nm) for Cy5-mRNA fluorescence.

### eGFP expression test by flow cytometry

To evaluate the translational efficiency of mRNA delivered by C14-PEI, we quantified the expression of the enhanced green fluorescent protein (eGFP) reporter gene using flow cytometry. H1299, A549, and Hop-62 cell lines were seeded at a density of 30 000 cells per well in 24-well plates containing 500 μL of growth medium. Following incubation in a cell culture incubator (37 °C, 5% CO_2_) for 24 h, the cells were transfected with C14-PEI micelleplexes encapsulating eGFP-mRNA at w/w8 and w/w 4 prepared at pH 7.4, and at a w/w ratio of 4 prepared at pH 5.4. PEI served as a control treatment. After 24 h of transfection, cells were washed with PBS and detached using 0.05% trypsin-EDTA. The detached cells were collected in 1.5 mL Eppendorf tubes and centrifuged at 300*g* for 5 minutes. The supernatant was aspirated, and cells were washed with PBS, followed by another centrifugation step. The cell pellet was resuspended in fresh PBS, and fluorescence intensity was measured using Attune NxT flow cytometry (Thermo Fisher, Germany) with excitation at 488 nm and emission at 510 nm. Results are presented as mean ± standard deviation (SD, *n* = 2 or 3).

### Agarose gel electrophoresis

Agarose gel electrophoresis was employed to confirm the co-encapsulation of Cas9 mRNA and sgRNA, as well as to perform the T7EI assay. A 1–1.5% agarose gel containing SYBR Safe (1 : 100 000 dilution) was prepared in TBE buffer. Subsequently, micelleplexes and free RNA samples, along with products from the T7EI assay, were mixed with 6× DNA loading dye and loaded onto the gel. Electrophoresis was conducted at 150 V for 40 minutes. The gel was visualised using the ChemiDoc imaging system (Bio-Rad, US).

### Editing efficiency test by T7EI assay

The T7EI assay was conducted according to the manufacturer's protocol using the GeneArt™ Genomic Cleavage Detection Kit. A549 cells were initially seeded in 6-well plates at a density of 100 000 cells per well in 1.5 mL of medium 24 h prior to the experiment. Following a media change, cells were transfected with C14-PEI micelleplexes containing Cas9 mRNA and sgRNA at w/w 8, w/w 4 prepared at pH 7.4, and w/w 4 prepared at pH 5.4. Lipofectamine 2000 was included as a positive control. Transfected cells were then incubated at 37 °C with 5% CO_2_ for 48 h. Subsequently, cells were washed with PBS, harvested using 0.05% trypsin-EDTA, and collected by centrifugation into 1.5 mL Eppendorf tubes. The cell pellets were lysed using lysis buffer, and the resulting lysates were utilised for PCR amplification of sequences containing *KRAS* alleles. Following PCR amplification, the PCR products underwent re-annealing and treatment with the detection enzyme as per the kit's instructions. Positive control samples provided in the kit, both with and without enzymes, were included for validation. Agarose gel electrophoresis was performed to visualize the cleavage products, and images were captured using the ChemiDoc imaging system as section 2.12 described. Data analysis was conducted using Image Lab Software.

### Droplet digital PCR

A549 cells were transfected in a 6-well plate using C14-PEI at w/w 4 and w/w 8 with Cas9 mRNA and sgRNA for 48 h, with lipofectamine 2000 used as a positive control. Genomic DNA was extracted from both untreated and treated A549 cells using the DNeasy Blood & Tissue Kit (Qiagen), and the DNA concentration was quantified using a Nanodrop spectrophotometer. Primers and probes were custom-designed and obtained from Bio-Rad. The reaction mixtures for ddPCR contained 2× ddPCR Supermix for Probes (no dUTP), with final concentrations of 900 nM for each primer and 250 nM for each FAM- or HEX-labelled probe. A total of 100 ng of template DNA was added to achieve a final reaction volume of 20 μL. Standard Bio-Rad reagents and consumables, including cartridges, gaskets, droplet generation oil, and droplet reader oil, were used. After droplet generation, droplets were carefully transferred to a 96-well PCR plate and sealed using the PX1 PCR Plate Sealer (Bio-Rad). The PCR conditions were as follows: initial denaturation at 95 °C for 10 minutes, followed by 40 cycles of denaturation at 94 °C for 30 seconds, annealing/extension at 55 °C for 3 minutes, and a final extension step at 98 °C for 10 minutes, followed by a hold at 4 °C. The ramp rate was set at 2 °C s^−1^. Droplets were read using the QX200 Droplet Reader (Bio-Rad), and each reaction included a no-template control (NTC). Data analysis was performed using QuantaSoft Software.^37^

### Sanger sequencing

Genomic DNA was extracted from A549 cells 48 h post-transfection with C14-PEI w/w 8 using the DNeasy Blood & Tissue Kit. To visualize the gene sequence after gene editing, PCR was performed using a pair of primers designed to target regions before and after the cleavage site, yielding a PCR product of approximately 500 base pairs. The Phusion Hot Start II High-Fidelity PCR Mastermix was utilised for PCR amplification. The cycling conditions were as follows: initial denaturation at 98 °C for 30 seconds, followed by 35 cycles of denaturation at 98 °C for 10 seconds, annealing at 61.5 °C for 30 seconds, extension at 72 °C for 30 seconds, and a final extension at 72 °C for 10 minutes. PCR products were verified by electrophoresis on 1% agarose gels. Following gel verification, PCR products were purified using the ExoSAP-IT™ Express PCR Product Cleanup Reagent. The purified PCR products were subsequently used for Sanger sequencing to determine the sequence changes resulting from the gene editing process. The results were analysed by the ICE CRISPR analysis tool.^38^

### Western blot

To assess the ability of C14-PEI micelleplexes to inhibit downstream signals in the KRAS pathway, A549 cells were seeded in 6-well plates and allowed to grow for 24 h to reach a density of 1 × 10^5^ cells per well. The cells were then treated with C14-PEI micelleplexes and incubated at 37 °C with 5% CO_2_ in a humidified incubator for 48 h. Following treatment, cells were washed with ice-cold PBS and lysed in lysis buffer consisting of 800 μL RIPA buffer, 100 μL Phosphatase inhibitor, and 100 μL Protease inhibitor. The protein content in the lysates was quantified using the Pierce™ BCA Protein Assay kit (Thermo Fisher), and equal amounts of protein were loaded for SDS-PAGE (Novex™ WedgeWell™ 8–16% Tris-Glycin gel). Separated proteins were then transferred onto nitrocellulose membranes, which were subsequently blocked with 5% skim milk in TBST (Tris-buffered saline with 1% Tween 20) for 1 h at room temperature. Membranes were then incubated overnight at 4 °C with primary antibodies targeting specific proteins of interest in the KRAS pathway. After primary antibody incubation, membranes were washed three times with 1% TBST and then incubated with horseradish peroxidase (HRP)-conjugated secondary antibodies at room temperature for 1 h. Protein bands were visualised using chemiluminescence substrates and imaged immediately using the ChemiDoc imaging system (BioRad, US). Between antibody stainings, membranes were treated with stripping buffer for 30 minutes to remove bound antibodies, followed by washing with TBST and re-blocking with 5% skim milk in TBST solution. This systematic approach allowed for the quantification of protein expression levels involved in the KRAS pathway inhibition following treatment with C14-PEI micelleplexes, providing insights into their therapeutic potential.^39^

### Wound healing assay

The μ-Dish with culture-insert 2 well (ibidi, Germany) was utilised for conducting a wound healing assay.^40^ Initially, 10 000 A549 cells suspended in 70 μL of RPMI-1640 media were added to each well of the Culture-Insert 2 Well and allowed to incubate at 37 °C with 5% CO_2_ for a minimum of 24 h to achieve a confluent cell layer. Following incubation, the insert was carefully removed using sterile tweezers, and the cell layer was washed twice with PBS to eliminate any cell debris and non-adherent cells. Subsequently, the μ-Dish was filled with 2 mL of fresh complete medium containing either C14-PEI micelleplexes or Lipofectamine 2000, as per experimental requirements. The cells were maintained in the incubator at 37 °C with 5% CO_2_ throughout the experiment, and images were captured at 0, 4, 8, 24, and 48 h using an EVOS microscopy (Thermo Fisher, Germany). The area of the wound gap was quantified and analysed using ImageJ software, providing insights into the migration and healing dynamics of the A549 cell monolayer in response to the treatments administered.

### Cell apoptosis

Annexin V and PI staining allowed for the quantification of apoptotic and necrotic cells, providing insights into the cellular response to C14-PEI micelleplex transfection.^41^ A total of 1 × 10^5^ cells per well were initially seeded onto a 6-well plate in RPMI-1640 complete medium and transfected with C14-PEI micelleplexes. Following a 48 h incubation at 37 °C with 5% CO_2_, the cells were washed twice with cold PBS and resuspended in Annexin V Binding Buffer at a concentration of 1 × 10^6^ cells per ml. Subsequently, 100 μL of the cell suspension was mixed with 10 μL of Annexin V-AF488 (Thermo Fisher) and 1 μL of PI (BD Biosciences), and the mixture was incubated for 15 minutes at room temperature in the dark. After incubation, 400 μL of Annexin V Binding buffer was added to each tube to halt the reaction. Fluorescence signals from Annexin V-AF488 and PI staining were measured using the Attune NxT flow cytometry (Thermo Fisher, Germany), and the data were analysed using FlowJo software.

### Statistics

Unless otherwise specified, all results are presented as the mean value ± standard deviation (SD) based on triplicate experiments (*n* = 3). Statistical analyses were performed using GraphPad Prism software (GraphPad Software, USA).

## Results

### C14-PEI synthesis and characterization

It has been reported that modifying polymeric micelleplexes with hydrophobic groups can enhance their affinity for cell membranes and improve the delivery of nucleic acids.^32,33^ Specifically, substituting free amines on cationic polymers with alkyl tails yields amphiphilic polymers, which promote particle formation through hydrophobic aggregation. Due to the hydrophobic nature of cell lipid bilayers, hydrophobic micelleplexes exhibit more favourable interactions with cell membranes.^42^ However, identifying suitable cationic polymeric carriers for mRNA delivery remains challenging due to mRNA's large molecular size, secondary structure, and intrinsic single-strand instability.^43^ Effective delivery of mRNA requires a carrier that balances stability, cellular uptake, and transfection efficiency. To achieve this, we synthesised C14-PEI, an amphiphilic polymer designed to enhance micelleplex formation and nucleic acid delivery through hydrophobic modification of PEI.

**Fig. 1:**
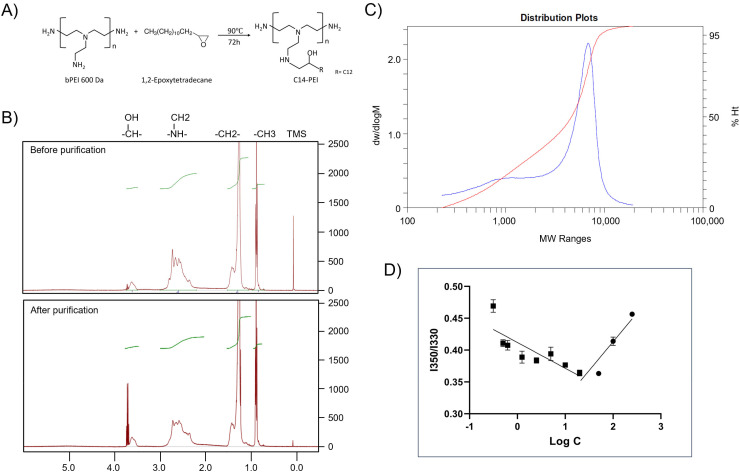
Characterization of C14-PEI. (A) The schematic of C14-PEI synthesis. (B) ^1^H NMR spectra of C14-PEI before and after dialysis. (C) The molecular weight of Mn 1697 Da of the final product (blue) was confirmed by GPC. (D) Pyrene fluorescence intensity at 350/330 as a function of polymer concentration (*n* = 3). CMC is noted as the point of inflection where fluorescence intensity begins to increase.

The synthesis of C14-PEI involved the reaction of 1,2-epoxytetradecane with bPEI 600 Da through a ring-opening reaction (Fig. 1A). The optimal degree of substitution was determined by varying the molar ratio of epoxy groups to total amines, and a 33% C14-PEI, corresponding to a 1 : 1 ratio of epoxy groups to primary amines, showed the highest eGFP mRNA transfection efficiency (Fig. S1†). Therefore, 33% C14-PEI was selected for subsequent experiments unless otherwise specified.

NMR was employed to evaluate the modification of C14-PEI before and after purification. The ^1^H NMR spectrum of C14-PEI exhibited characteristic signals corresponding to both the PEI backbone and the pendant carbon strand moieties (Fig. 1B). The PEI backbone displayed major peaks at *δ* 2.2–3.8 parts per million (ppm). Additionally, characteristic proton peaks influenced by the hydrophobic moieties were observed at *δ* 3.5–3.75 ppm. Strong signals at 0.75 and 1.25 ppm were attributed to the methyl and alkyl groups on the carbon strands, respectively. Importantly, the ^1^H NMR analysis revealed the complete disappearance of the epoxy group, confirming the absence of free 1,2-epoxytetradecane starting material. In comparison to unpurified C14-PEI, the purified sample exhibited similar chemical shifts for the main functional groups but with notable improvements: reduced noise, sharper peaks, better resolution, and decreased overlap. These characteristics collectively indicated a purer sample with successful removal of impurities during the purification process.

The molecular weight of the final product, determined by GPC, was *M*_n_ 1700 Da (Fig. 1C), consistent with theoretical calculations (*M*_n_ 1600–1800 Da) based on the modification of primary amines at a 1 : 1 molar ratio. The polydispersity index (PDI) of the final product was 2.6, indicating a more uniform polymer distribution compared to the unpurified polymer (PDI: 3.16, Fig. S3†), which is advantageous for subsequent drug delivery applications.

Due to its amphiphilic nature, C14-PEI forms micellar structures in aqueous media. The CMC was determined using pyrene as a probe molecule, based on its linear relationship with polymer concentration.^34^ The CMC of C14-PEI was measured at 20.86 ± 0.15 mg L^−1^ (Fig. 1D), which was lower than small-molecule surfactants, supporting stability of micelles and potential protection from opsonization *in vivo*.^36^

### Preparation and characterization of C14-PEI micelleplexes

**Fig. 2:**
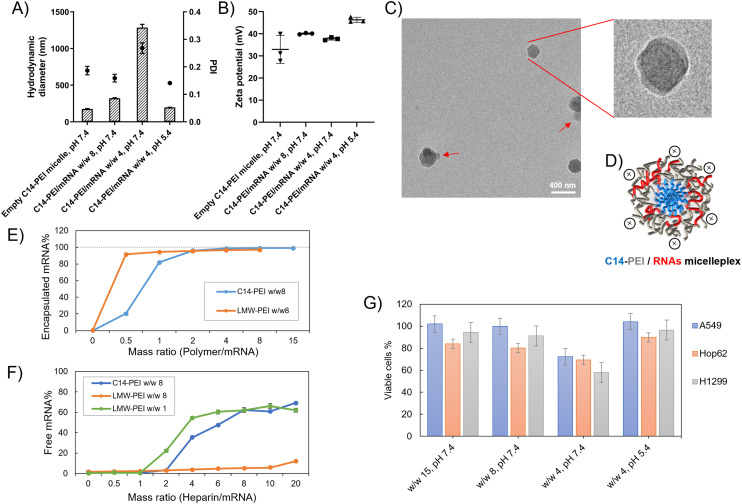
Characterization of C14-PEI formulation. (A) the hydrodynamic diameter (bars) and polydispersity index (PDI, dots) of micelleplexes (*n* = 3); (B) the zeta potential of micelleplexes (*n* = 3); (C) Cryo-EM image of C14-PEI w/w 8; (D) schematic drawing of C14-PEI w/w 8; (E) SYBR Gold assay to assess the encapsulation and (F) heparin competition assay to determine release (*n* = 3); (G) the percentage of viable cells after 24 h transfection in A549 cells (*n* = 3).

Micelleplexes were prepared by complexing C14-PEI with eGFP mRNA at varying mass ratios to identify optimal formulation parameters. Based on flow cytometry screening results (Fig. S2†), 33% C14-PEI at a w/w of 8 at pH 7.4 and w/w 4 at pH 7.4 were chosen for further testing. Additionally, a formulation at w/w 4 prepared at pH 5.4 was selected for comparison. To characterize the micelleplexes, DLS and LDA were employed to measure their size and zeta (*ζ*) potential. Empty C14-PEI micelles at the same concentration as w/w 8 micelleplexes were analysed to assess their properties. The empty micelles exhibited a hydrodynamic diameter of 175 nm with a PDI of 0.19 and a zeta potential of 33 mV (Fig. 2A and B). Upon mRNA loading, the w/w 8 micelleplexes showed an increased size of approximately 300 nm with a PDI of 0.16. At a lower w/w ratio of 4, the micelleplexes exhibited significant aggregation, resulting in a particle size exceeding 1000 nm and a PDI of 0.27. Interestingly, reducing the pH to 5.4 for the w/w 4 formulation decreased the size to 200 nm (PDI: 0.14), likely due to enhanced electrostatic interactions. Despite these differences in size and aggregation, all three micelleplex groups maintained zeta potentials in the range of 40–45 mV. This moderately positive zeta potential suggests adequate electrostatic repulsion, crucial for preventing particle aggregation and maintaining stability in suspension.^44^ Given that most cellular membranes are negatively charged, positively charged nanoparticles may facilitate enhanced cellular uptake through strong interactions. However, cationic particles are generally associated with increased toxicity due to potential cell membrane disruption.^45^ Moreover, an excessively positive zeta potential could lead to over-stabilization, potentially affecting drug release profiles.^44^ Therefore, careful consideration of particle stability, toxicity, and cellular uptake is essential for subsequent experiments.

The C14-PEI delivery system tends to form micelleplexes, where the hydrophobic chains constitute the core and the hydrophilic portions form the shell. Cryo-EM imaging confirmed the presence of a core–shell spherical structure in these micelleplexes. Fig. 2C displays the particle sizes of C14-PEI micelles at w/w 8, pH 7.4, which align with the DLS results, showing sizes around 300 nm. In the images, a distinct dark core is surrounded by a blurred corona structure. Additionally, in Fig. 2C, bleb-like structures are observed in the nanostructures. Similar bleb structures have been reported in lipid nanoparticles (LNPs) by Cheng and colleagues.^46^ They noted that alterations in pH and buffer concentration could induce the formation of these structures, thereby enhancing *in vitro* transfection efficiency. This enhancement is attributed to improved mRNA stability when sequestered within bleb structures in LNPs.

### The assessment of the formulation properties

#### Encapsulation

As previously noted, mRNA is inherently unstable due to its single-stranded structure, making it susceptible to degradation by nucleases.^47^ Protecting mRNA from nuclease digestion is crucial, and the ability of polymers to encapsulate mRNA is a key factor in assessing their suitability as mRNA carriers. SYBR Gold, a fluorescent intercalating dye that stains free nucleic acids and emits fluorescence upon excitation at 495 nm, is commonly used for this purpose.^48^ Cationic polymers interact electrostatically with the negatively charged phosphate groups present in mRNA molecules, facilitating the encapsulation of mRNA within micelleplexes through charge complexation. This interaction decreases accessibility for intercalation and thus reduces the fluorescence intensity of SYBR Gold, allowing for the quantification of free mRNA in the micelleplex suspensions by measuring fluorescence intensity.

Fig. 2E illustrates the use of LMW-PEI as a control, where fluorescence measured with free mRNA was established as 100%. The percentage of free mRNA decreased with increasing the formulations’ mass ratio, indicating polymer-mediated encapsulation of mRNA into polyelectrolyte complexes. Below a mass ratio of w/w 2, C14-PEI demonstrated less efficient mRNA condensation compared to LMW-PEI. Specifically, C14-PEI exhibited only 20% encapsulation efficiency at w/w 1, whereas LMW-PEI efficiently encapsulated mRNA even at very low mass ratios. However, at a mass ratio of w/w 2, the encapsulation efficiency of C14-PEI began to approach that of LMW-PEI, showing approximately 4% free mRNA. As the mass ratio increased further, C14-PEI demonstrated superior condensation capability compared to LMW-PEI, achieving mRNA encapsulation efficiencies of around 98% and nearly 100% at w/w 4 and w/w 8, respectively. This behaviour can be attributed to the occupation of primary amine groups by C14 chains, which reduces positive charges. Initially, at low mass ratios, C14-PEI exhibited lower mRNA encapsulation efficiency compared to LMW-PEI. However, with increasing polymer concentration, as observed elsewhere,^49^ amphiphilic materials condense mRNA through electrostatic and hydrophobic interactions, demonstrating high nucleic acid-binding affinity.

### mRNA release

The stability of micelleplexes can be disrupted by the presence of competing anions.^50^ To assess the stability of C14-PEI/mRNA complexes and gain deeper insights into micelleplex behaviour, we investigated the integrity of micelleplexes in the presence of a competing polyanion (heparin) using SYBR Gold staining. LMW-PEI was included as a control at polymer to mRNA mass ratios of w/w 1 and w/w 8. In Fig. 2F, at the w/w 8 polymer/mRNA mass ratio, mRNA remained tightly bound to LMW-PEI even with a 20-fold excess of heparin relative to mRNA (heparin/mRNA w/w 20, approximately 2 units of heparin). This strong binding is attributed to the excessive positive charges in PEI, which result in a robust interaction with mRNA, hampering its release. While stable complexes in the presence of competing anions are desirable, overly strong binding can hinder mRNA release from micelleplexes into the cytoplasm.^51^ Reducing the amount of LMW-PEI improved the situation; at a w/w 1 polymer/mRNA mass ratio, the LMW-PEI/mRNA complexes remained stable until the heparin to mRNA ratio reached 1 unit (heparin/mRNA w/w 1).

In contrast, C14-PEI w/w 8 exhibited a release profile similar to LMW-PEI at w/w 1. mRNA began to release from C14-PEI at a heparin to mRNA mass ratio of w/w 2. Notably, the micelleplexes demonstrated stability in the presence of up to a 2-fold excess of heparin/mRNA and released approximately 70% of mRNA at a 20-fold excess of heparin (Fig. 2F). Optimizing polymer concentrations advantageously contributes to micelleplex stability after introduction into serum-containing cell culture media or administration *in vivo*, and facilitates mRNA release from micelleplexes into the cytoplasm.

#### Cytotoxicity

A significant drawback of cationic delivery systems is their potential toxicity arising from high positive charge densities, which can disrupt cellular membrane integrity and lead to pore formation.^52^ To assess cytotoxicity, the CCK-8 assay was employed, which measures the intracellular reduction of tetrazolium salt (WST-8) to produce an orange water-soluble formazan dye through bioreduction in the presence of an electron carrier, 1-Methoxy PMS. The absorbance of this dye correlates linearly with the number of viable cells, providing a direct measure of toxicity. Cytotoxicity testing was conducted using different cell lines, A549, H1299, and Hop62. C14-PEI/mRNA complexes at various mass ratios (w/w 15, w/w 8, w/w 4 prepared at pH 7.4, and w/w 4 prepared at pH 5.4) were evaluated. After 24 h of transfection, all groups exhibited low toxicity across the three cell lines, except for the w/w 4 group prepared at pH 7.4 (Fig. 2G). Specifically, cell viability with C14-PEI at w/w 15 and w/w 8 prepared at pH 7.4 exceeded 80%. In contrast, the w/w 4 group prepared at pH 7.4 displayed higher toxicity, resulting in less than 60% cell viability. Interestingly, reducing the pH to 5.4 mitigated toxicity in the w/w 4 group, achieving comparable cell viability (around 80%) to those observed in the w/w 15 and w/w 8 groups prepared at pH 7.4. This observation is consistent with the tendency of particles to aggregate at w/w 4 at pH 7.4, as indicated by DLS results.

### Delivery of mRNA

#### Endosomal escape test

**Fig. 3:**
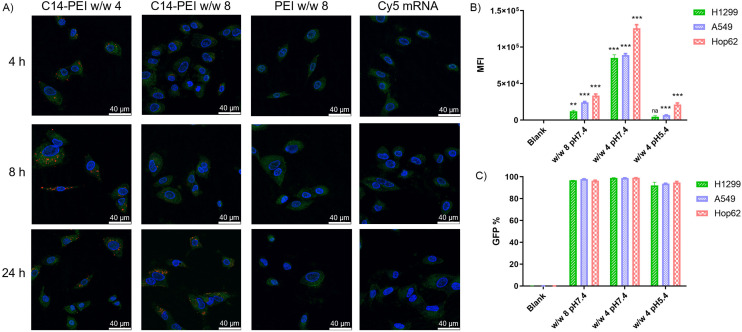
Delivery of eGFP mRNA. (A) Endosomal entrapment of C14-PEI formulation *via* CLSM. (B) Median eGFP fluorescence intensity after C14-PEI transfection 24 h (*n* = 3, *P ≤ 0.033, **P ≤ 0.002, ***P ≤ 0.001). (C) The percentage of eGFP positive cells.

The endocytic pathway is the major uptake mechanism for nanocomplexes.^53^ Micelleplexes become entrapped in endosomes and are subsequently degraded by specific enzymes in lysosomes. Therefore, facilitating endosomal escape to ensure the cytosolic delivery of therapeutics is a critical step in achieving effective macromolecule-based therapy.^54^ To accurately depict the endosomal release of micelleplexes internalised by cells, A549 cells were transfected with Cy-5 mRNA and stained with LysoTracker Green DND-26, a fluorescent probe that accumulates in acidic vesicles, along with DAPI staining before analysis by CLSM. Fig. 3A illustrates different formulation treatments and their effects on cellular uptake and endosomal release of Cy-5 labelled mRNA. Blue areas depict cell nuclei stained with DAPI, green staining indicates lysosomes, red staining represents incorporated Cy-5 labelled mRNA, and yellow dots reflect mRNA co-localised within lysosomes.

As shown in the Fig. 3A, no red signal is detected in samples with free mRNA or PEI formulation, indicating that successful uptake cannot occur without an appropriate delivery system. However, C14-PEI transfection at w/w 4 and w/w 8 results in a punctate distribution of Cy-5 labelled mRNA, suggesting endosomal entrapment of the delivered cargo. Despite the persistence of yellow dots after 24 h, indicating partial entrapment in endosomes, a significant proportion of mRNA was able to escape and disperse into the cytoplasm. Specifically, at 4 h post-transfection, only a few red and yellow dots are observed in both the w/w 4 and w/w 8 groups, reflecting early cellular internalization. By 8 h, maximum uptake is observed in the C14-PEI w/w 4 samples, with the signal decreasing by 24 h. In contrast, uptake of C14-PEI at w/w 8 continues to increase, reaching a maximum at 24 h. Interestingly, Fig. 3A shows that the red dots, representing Cy-5 labelled mRNA, were more enriched and appeared larger in the w/w 4 samples compared to the w/w 8 samples. This suggests that C14-PEI micelleplexes were more prone to aggregation at the w/w 4 ratio, which aligns with the larger sizes observed in the DLS results. Consequently, these aggregated micelleplexes exhibited faster sedimentation and higher sedimentation efficiency in the cell media. This resulted in faster internalization of C14-PEI at w/w 4 compared to C14-PEI at w/w 8.

#### eGFP mRNA expression

To investigate the mRNA expression efficacy of the C14-PEI formulation, eGFP mRNA was transfected into H1299, A549, and Hop62 cell lines using formulations of w/w 8, w/w 4 prepared at pH 7.4, and w/w 4 prepared at pH 5.4. Following transfection, median fluorescence intensity (MFI) and the percentage of eGFP-positive cells were measured using flow cytometry (FACS). As shown in Fig. 3C, all groups exhibited eGFP expression, with over 90% of cells in all three cell lines being eGFP-positive. The w/w 4 formulation prepared at pH 7.4 resulted in an MFI that was over 1000-fold higher than the blank control (Fig. 3B). The lowest MFI was observed with the w/w 4 formulation prepared at pH 5.4, which showed a 30–40 fold increase compared to the blank. The w/w 8 formulation achieved an MFI increase of beyond 100-fold compared to the blank. The significantly high eGFP expression observed in the C14-PEI w/w 4 group at pH 7.4 can be attributed to rapid and efficient cellular internalization.

### Co-encapsulation of Cas9 mRNA and sgRNA

**Fig. 4:**
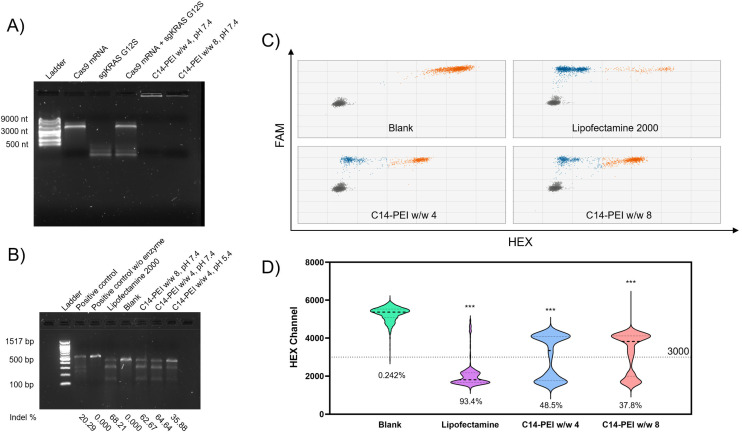
Co-delivery of Cas9 mRNA and sgRNA. (A) Agarose gel shows the co-encapsulation of Cas9 mRNA and sgRNA in C14-PEI formulation. (B) T7EI cleavage tests in agarose gel, gene editing efficiency is indicated below the image. (C) Droplet distribution of ddPCR, *X*-axis is HEX channel, *Y*-axis is FAM channel, Gray dots represent the FAM-negative/HEX-negative group, orange dots represent the FAM-positive/HEX-positive group, blue dots represent the FAM-positive/HEX-negative group. (D) Violin plots of the HEX channel (excludes FAM negative droplets) of ddPCR (***P ≤ 0.001), intensity at 3000 is set as threshold, and editing efficiency is indicated below the plots.

C14-PEI/Cas9 mRNA-sgRNA micelleplexes were prepared using the previously described method and characterised with a Nanosizer (Fig. S4†). These micelleplexes, co-loaded with Cas9 mRNA and sgRNA, displayed properties similar to those of eGFP mRNA micelleplexes. This similarity arises from the consistent polymer-to-total RNA ratios employed during preparation. As both sgRNA and mRNA are single-stranded RNAs, their total mass corresponds to their nucleotide content, resulting in comparable physicochemical properties between the two formulations.^55–57^ Given that the efficiency of this CRISPR gene editing approach relies on the successful delivery of both mRNA and sgRNA, confirming their co-encapsulation is essential. Electrophoretic mobility shift assays (EMSA) were employed for this evaluation, leveraging the ability of electrophoresis to separate nucleic acid molecules of different sizes by electrophoretic mobility.^58^ In these assays, free RNA migrates through the gel due to its negative charge, whereas encapsulated RNA remains in the loading slots, as the micelleplexes are larger than the gel's mesh size.^59^ As shown in Fig. 4A, lanes 2 and 3 display the free Cas9 mRNA and free sgRNA bands, located at 4500 nt and 100 nt, respectively. Lane 4 clearly shows the separated bands of the mixture of free Cas9 mRNA and sgRNA. In contrast, lanes 5 and 6, containing samples of C14-PEI w/w 4 and w/w 8, show no bands on the gel. Instead, a bright signal is visible within the slots, indicating that both Cas9 mRNA and sgRNA are encapsulated within the micelleplexes.

### Evaluation of CRISPR gene editing ability

#### T7EI assay

To further verify the ability of the C14-PEI formulation to co-deliver Cas9 mRNA and sgRNA and facilitate gene editing in cells, we transfected A549 cells, harbouring a *KRAS* G12S mutation,^60^ with Cas9 mRNA and sgRNA targeting the *KRAS* G12S allele using C14-PEI micelleplexes. Numerous methods for verifying CRISPR gene editing have been reported in the literature.^37,61–63^ Due to the sensitivity limitations of experimental and analytical methods, a single detection method cannot accurately reflect gene knockout efficiency. We performed three different assays to evaluate the deletion of *KRAS* G12S alleles in A549 cells. Specifically, genomic DNA was isolated from transfected cells 48 h post-transfection. The T7EI assay, ddPCR, and Sanger sequencing were then conducted to measure gene editing efficiency.

Non-homologous end joining (NHEJ) is the primary mechanism for knockout mediated by CRISPR-Cas9.^64^ During NHEJ, insertions and/or deletions (Indels) are commonly induced in the DNA strand. T7 Endonuclease I, a structure-selective enzyme, specifically recognizes indel sites on the DNA sequence and cleaves them into two fragments.^63^ The digestion products can then be visualised and analysed by agarose gel electrophoresis.

As shown in Fig. 4B, the untreated sample exhibited only one band corresponding to the target sequence, while all treated samples displayed both the mother band and two cleaved bands. From the intensity analysis, the Lipofectamine 2000 group resulted in 68.21% indels. Similarly, the w/w 8 and w/w 4 formulations prepared at pH 7.4 mediated an average of 62.67% and 64.64% indels, respectively, demonstrating comparable gene editing efficiency to the Lipofectamine 2000 group. However, the cells treated with the w/w 4 formulation prepared at pH 5.4 exhibited only 35.88% indel formation, consistent with the eGFP expression results.

#### ddPCR analysis

To further confirm the gene editing, the number of gene copies was measured using ddPCR to quantify NHEJ-mediated events in the samples. In this assay, two specific probes within one amplicon were designed.^37,62^ The first probe, a reference probe (FAM), is located away from the mutagenesis site and counts all genomic copies of the target. The second probe, an NHEJ probe (HEX), is located at the site where nucleases cut or nick genomic DNA and has a wild-type (WT) sequence. If nucleases induce NHEJ, the NHEJ probe loses its binding site, resulting in the loss of the HEX signal and leaving only the FAM signal from the reference probe. As shown in Fig. 4C, the orange group indicates FAM and HEX double-positive droplets, reflecting WT DNA copies, while the blue group shows FAM-positive but HEX-negative droplets, representing edited DNA copies. No blue dots are present in the blank group, while the groups treated with C14-PEI w/w 4 and w/w 8 show 961 and 1430 blue dots respectively (edited gene copies), indicating efficient gene editing events. Subsequently, we quantified the percentage of single-positive events (edited gene copies) in the total events. As shown in Fig. 4D, the gene editing efficiency of Lipofectamine 2000 reached 93.4%, whereas C14-PEI w/w 4 showed 48.5% edited copies and 37.8% positive droplets in the C14-PEI w/w 8 group. The data from ddPCR did not align perfectly with the T7EI assay results. This discrepancy arises because the T7EI assay is semi-quantitative, has limited sensitivity, is prone to false positives, and suffers from high background signals when sequence polymorphisms are present.^63^ For a typical diploid target locus, a clone with both alleles successfully altered *via* genome editing will be indistinguishable from a clone with one mutated allele and one wild-type allele.

#### Sanger sequencing

**Fig. 5:**
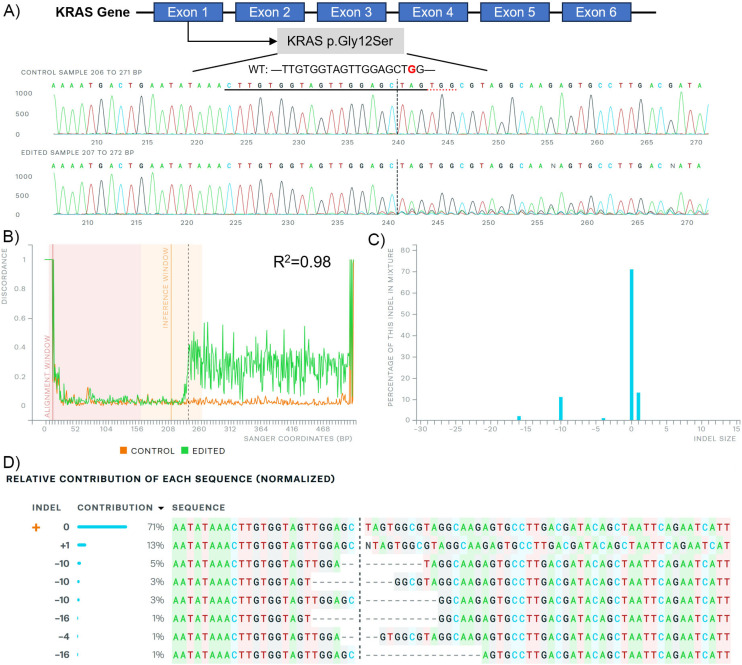
Sanger sequencing after C14-PEI w/w 8 treatment in A549 cells analysed by the ICE CRISPR analysis tool. (A) KRAS exon map (up), G12S mutation sequence (middle), and edited sequence (down) illustrate; (B) alignment of Sanger sequencing; (C) distribution of indel sizes; (D) contribution of each sequence after gene editing.

To visualize the gene editing behaviour of the C14-PEI w/w 8 micelleplexes, we performed Sanger sequencing on the PCR products and analysed the data using the ICE CRISPR analysis tool.^38^Fig. 5D demonstrates that indels occurred in the *KRAS* G12S allele edited by C14-PEI w/w 8. Sequencing confirmed that gene editing occurred after the PAM sequence, primarily resulting in insertions and deletions in the DNA backbones. Specifically, Fig. 5B shows a significant signal shift (*R*^2^ = 0.98) following gene editing compared with the control sequence. Among the generated mutations (Fig. 5C and D), a 1 bp insertion was the most frequent, contributing to 13% of the indels, which aligns with previously reported findings.^60^ Deletions ranging from 4 to 16 bases were found at various positions near the mutagenesis site, constituting 14% of the indels. These indels cause frameshift mutations in the gene, leading to the functional inactivation of the mutant KRAS protein. In summary, the sequencing data confirmed that *KRAS* in A549 cells was disrupted around the PAM (TGG) sequence, further validating the efficacy of our C14-PEI delivery system in achieving efficient and specific targeting of *KRAS* G12S alleles.

### Cell capability assessment

#### Western blot

**Fig. 6:**
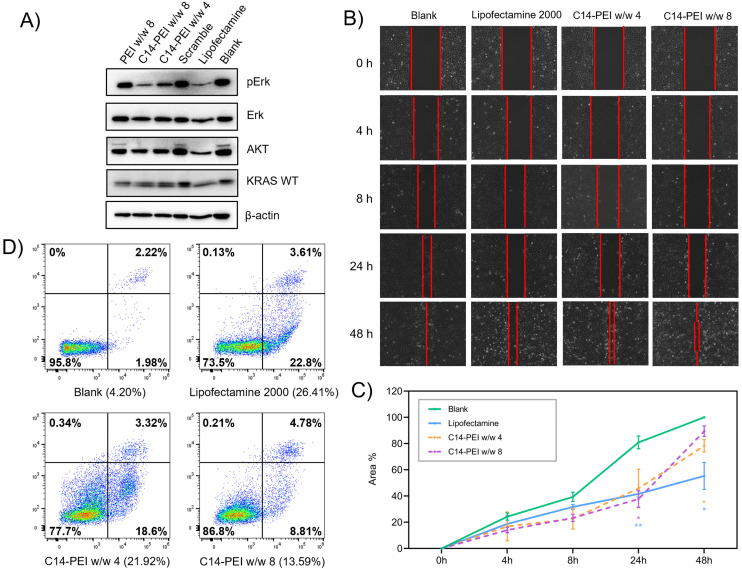
Cell capability assessment after the transfection of C14-PEI micelleplexes. (A) Western blot after transfection 48 h in A549 cells; (B) images of A549 cells in the wound healing assay in 48 h after transfection; (C) the line graph shows the average percentages with SD (*n* = 3) of covered area in wound healing assay (*P ≤ 0.0332, **P ≤ 0.021, the significance against the blank group); (D) cell apoptosis after transfection 24 h in A549 cells.

The *KRAS* gene mediates the translation of the KRAS protein, which relays signals from outside the cell to the nucleus. KRAS is a small GTPase that cycles between the GTP-bound active state and the GDP-bound inactive state. In its GTP-bound state, KRAS interacts with and activates downstream effector molecules, such as those in the MAPK or AKT-mTOR signalling pathways, affecting cell proliferation and survival. However, activating mutations in KRAS result in impaired GTP hydrolysis or enhanced nucleotide exchange, causing continuous downstream signal activation. This leads to a sustained proliferation signal within the cell, which is related to the migration and invasion of cancer cells.^65,66^ To assess the translation level of different signal proteins, we isolated total proteins from transfected A549 cells and conducted western blot analysis to investigate if the C14-PEI formulation can down-regulate KRAS pathways on the protein level, including the expression and activation of AKT and ERK. PEI and Lipofectamine 2000 were used as controls. As shown in Fig. 6A, compared to the housekeeping gene β-actin, the treatment of A549 cells with C14-PEI w/w 8 did not suppress the expression of wild-type KRAS protein. However, the level of phosphorylated-ERK protein was significantly downregulated in A549 cells edited with the micelleplexes. According to the literature,^67^ phosphoproteins usually will have a minor shift in molecular weight and total antibodies can recognize them. Hence, the upper bands in the AKT blot were deemed to represent phosphorylated-AKT, fitting the expectation of downregulation in the treated groups. As predicted, total AKT and ERK proteins were not affected by the treatment. These results suggest that while the overall levels of KRAS, AKT, and ERK proteins remain unchanged, the downstream signalling pathways, particularly those involving phosphorylated forms of AKT and ERK, are downregulated in cells treated with the C14-PEI micelleplexes. This indicates the potential effectiveness of the C14-PEI delivery system in mitigating the aberrant signalling caused by mutant *KRAS*, thereby affecting cell proliferation and survival pathways involved in cancer progression.

#### Cell migration

A wound healing assay was used to estimate the ability of cell migration after treatment with C14-PEI micelleplexes in A549 cells. The gap area closure was quantified by comparing images from time 0 h to 48 h using ImageJ. Generally, the treated groups showed slower cell migration compared to the blank group, and the group treated with Lipofectamine 2000 exhibited the slowest migration among all groups (Fig. 6C and D). After 8 h, the blank group migrated and covered approximately 50% of the wound area quantified at time 0 h. Meanwhile, the C14-PEI w/w 8 group showed the lowest coverage of the wound area, occupying around 20%, while cells treated with C14-PEI w/w 4 and Lipofectamine 2000 covered nearly 30% of the wound area. After 48 h, the wound in the blank group was completely closed, and the coverage of the wound area in the C14-PEI w/w 4 and w/w 8 groups grew to 85% and 92%, respectively. Only 70% of the wound area was covered in the Lipofectamine 2000 group at 48 h. Given that Lipofectamine 2000 is known to exhibit high cytotoxicity,^68^ the slowest migration observed in this group is expected. However, since our C14-PEI formulation did not show significant cytotoxicity (Fig. 2G), the results are reliable and suggest that the C14-PEI micelleplexes can effectively inhibit tumour cell migration.

#### Cell apoptosis

To further investigate whether C14-PEI micelleplexes inhibited A549 cell growth through the induction of apoptosis, the percentage of apoptotic cells was assessed using flow cytometry with Annexin V-AF488/PI double-staining assay following treatment with C14-PEI w/w 4 and w/w 8.^41^ Lipofectamine 2000 was included as a positive control. The representative flow cytometry data are presented in Fig. 6D. It was demonstrated that treatment with C14-PEI w/w 4, C14-PEI w/w 8, and Lipofectamine 2000 for 24 h significantly increased the numbers of apoptotic cells compared with the blank group. Notably, Lipofectamine 2000 exhibited the highest percentage of apoptotic cells (26.41%), followed by C14-PEI w/w 4 (21.92%) and C14-PEI w/w 8 (13.59%).

Protein translation and cell function don't always show a consistent tendency with gene editing, because of incomplete knockout efficiency and functional compensation.^69^ When a gene is edited or knocked out, cells can activate alternative pathways to compensate for the loss of function. This can involve the upregulation of genes with similar functions or the activation of parallel pathways to maintain cellular homeostasis. In particular, genes involved in the cell cycle and DNA repair were identified as essential, suggesting compensatory mechanisms when these pathways are disrupted.^70^ However, our results suggested that the deletion of mutant *KRAS* G12S alleles by C14-PEI micelleplexes can effectively inhibit tumour cell proliferation and migration, and promote the apoptosis of tumour cells after treatment, likely through downregulation of the AKT and ERK signalling pathways. These findings further support the potential of C14-PEI micelleplexes as a delivery system for gene editing and other therapeutic applications.

## Conclusions

CRISPR-Cas9 has emerged as a highly effective and customizable tool for genome editing, holding promise for the treatment of *KRAS* mutations in lung cancer,^60,71,72^ however, developing an efficient and bio-safe material is a key barrier. In this study, we introduce C14-PEI as a micelleplex system capable of efficiently co-delivering Cas9 mRNA and sgRNA to excise mutated *KRAS* alleles in lung cancer cells. C14-PEI is synthesised from 1,2-epoxytetradecane and branched PEI 600 Da *via* ring-opening reaction, exhibiting a CMC of 20.86 ± 0.15 mg L^−1^. Effective condensation of mRNA *via* electrostatic interaction was demonstrated across all tested polymers, even at low concentrations. Specifically, C14-PEI at w/w of 4 and 8, under pH 7.4 conditions, as well as at w/w 4 under pH 5.4, were selected for detailed investigation based on mRNA expression levels, particle size, and material toxicity considerations. Optimal conditions were identified with C14-PEI at w/w 8 and pH 7.4, revealing micelleplexes with a hydrodynamic diameter of approximately 300 nm (PDI: 0.16) and a zeta potential of 40 mV. Notably, C14-PEI at w/w 8, pH 7.4, exhibited stable complex formation under physiological conditions as confirmed by the heparin competition assay, along with efficient endosomal escape properties intracellularly. Encapsulation efficiency of eGFP mRNA by C14-PEI reached 99% at w/w 8, resulting in a 130-fold increase in expression compared to the blank control. These findings underscore C14-PEI's potential as a robust delivery system for CRISPR-Cas9 components, highlighting its suitability for targeted genome editing applications in cancer therapy. The study revealed that C14-PEI micelleplexes effectively co-deliver Cas9 mRNA and sgRNA for targeted genome editing of *KRAS* mutations in lung cancer cells. Notably, while C14-PEI at a w/w 4 and pH 7.4 exhibited the highest eGFP expression (>1000-fold increase), it also displayed larger particle sizes (>1000 nm) and increased cytotoxicity in the CCK-8 assay. This phenomenon was attributed to aggregation, as confirmed by CLSM during endosomal escape testing. Agarose gel analysis confirmed efficient co-encapsulation of Cas9 mRNA and sgRNA by C14-PEI micelleplexes. For gene editing purposes, at a sgRNA to Cas9 mRNA molar ratio of 10, C14-PEI micelleplexes demonstrated successful excision of the *KRAS* mutant with 62.67% and 64.64% indel efficacy at w/w 8 and w/w 4 pH 7.4, respectively. ddPCR further confirmed edited gene copies at 37.8% and 48.5% for w/w 8 and w/w 4 prepared at pH 7.4, respectively. Deletions and insertions under 16 base pairs were predominant in the edited gene sequences, as revealed by Sanger sequencing analysis. Following the deletion of *KRAS* G12S in A549 cells, downstream signalling was attenuated, as evidenced by decreased levels of phosphorylated-AKT and phosphorylated-ERK observed in western blot analysis. Moreover, the migration capability of A549 cells was impaired, and apoptosis was increased following treatment with C14-PEI micelleplexes containing Cas9 mRNA and sgRNA targeting *KRAS* G12S. These findings underscore the potential of C14-PEI as an efficient and relatively safe delivery system for CRISPR-Cas9-mediated genome editing, with implications for therapeutic interventions targeting *KRAS* mutations in lung cancer.

In conclusion, this study highlights the continuous evolution and potential of C14-PEI micelleplexes in advancing CRISPR-Cas9-based therapies for targeted genetic interventions, particularly in addressing mutations such as *KRAS* in cancer treatment. Leveraging the straightforward synthesis and functional groups of C14-PEI polymers, adjustments in chemical and physical properties can readily be made to enhance their efficacy as mRNA delivery agents and optimize their performance. This research affirms that hydrophobic modification of cationic polymers, C14-PEI, is conducive to designing drug delivery systems with improved cellular internalization capabilities and minimal toxicity. However, challenges remain, particularly concerning the size and zeta potential of micelleplexes, which may elicit immune responses and compromise efficiency *in vivo*. Addressing these issues is crucial for advancing toward clinical applications, and future efforts will focus on polymer modifications and composition adjustments to optimize micelleplex properties. Ongoing studies also aim to incorporate anionic polymers into the optimised formulations to further tailor nanoparticle characteristics and enhance therapeutic outcomes.

## Author contributions

Siyu Chen: data curation, investigation, methodology, and writing – original draft. Mariem Triki: investigation and methodology. Simone P. Carneiro: methodology, project administration, supervision, and writing – review. Olivia M. Merkel: conceptualization, funding acquisition, supervision, and writing – review and editing.

## Data availability

All data generated or analysed during this study are included in the main manuscript and in the ESI.†

## Conflicts of interest

There are no conflicts to declare.

## Supplementary Material

NR-017-D4NR03471F-s001
