# Supplementary material for: A novel micelleplex for tumour-targeted delivery of CRISPR-Cas9 against KRAS-mutated lung cancer
Source: Nanoscale. 2025 Jan 13;17(11):6604–19. doi: 10.1039/d4nr03471f (PMC11751667; doi:10.1039/d4nr03471f)
Supplement: NR-017-D4NR03471F-s001 [file NR-017-D4NR03471F-s001.pdf]

Supplementary Information (SI) for Nanoscale.
This journal is © The Royal Society of Chemistry 2025

A Novel Micelleplex for Tumour-Targeted Delivery of CRISPR-Cas9 against KRAS-Mutated Lung Cancer

Siyu Chen, [a] Mariem Triki, [a] Simone Pinto Carneiro, [a] and Olivia Monika Merkel *[a]
a Ludwig-Maximilians-University, Department of Pharmacy, Pharmaceutical Technology and Biopharmaceutics, Butenandtstraße 5-13, Munich, 81377, Germany

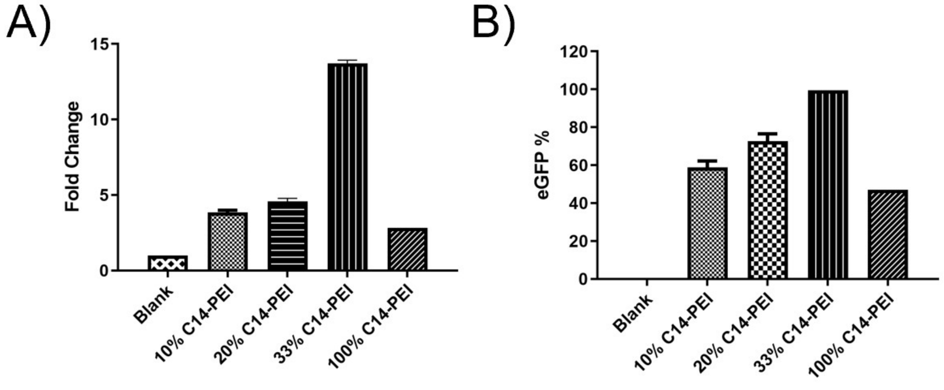

Figure S1. eGFP mRNA expression with C14-PEI at different modification level in A549 cells. A) Median eGFP fluorescence intensity after transfection 24h, results are normalised fold change; **B)** the percentage of eGFP positive cells.

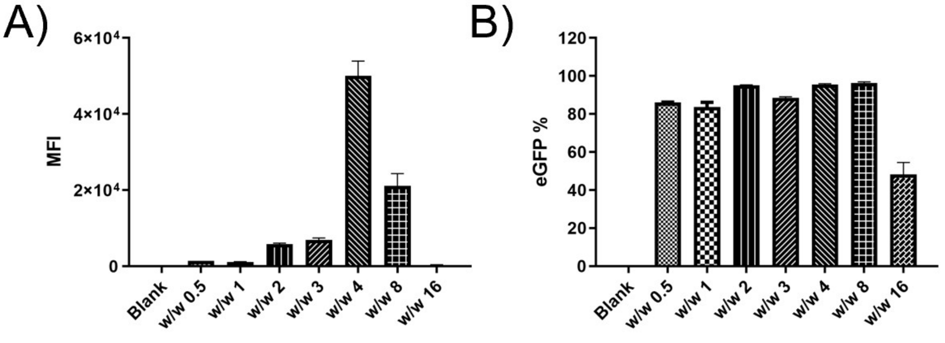

Figure S2. eGFP mRNA expression with 33% C14-PEI at different mass ratios in A549 cells. A) Median eGFP fluorescence intensity after transfection 24h; **B)** the percentage of eGFP positive cells.

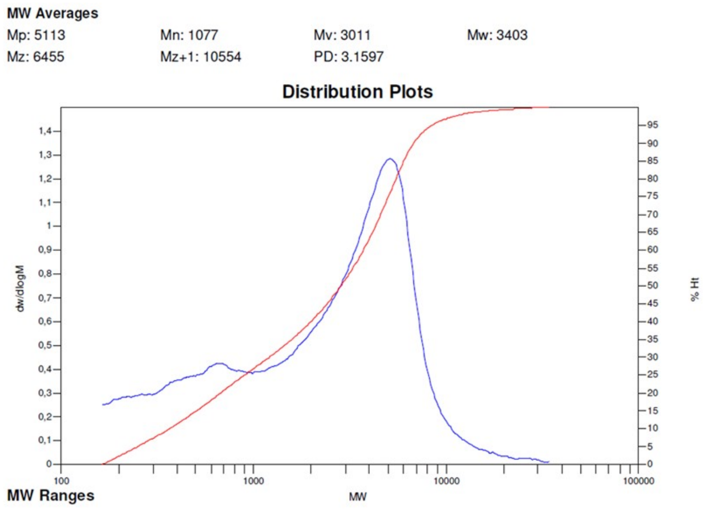

Figure S3. GPC analysis of 33% C14-PEI before purification.

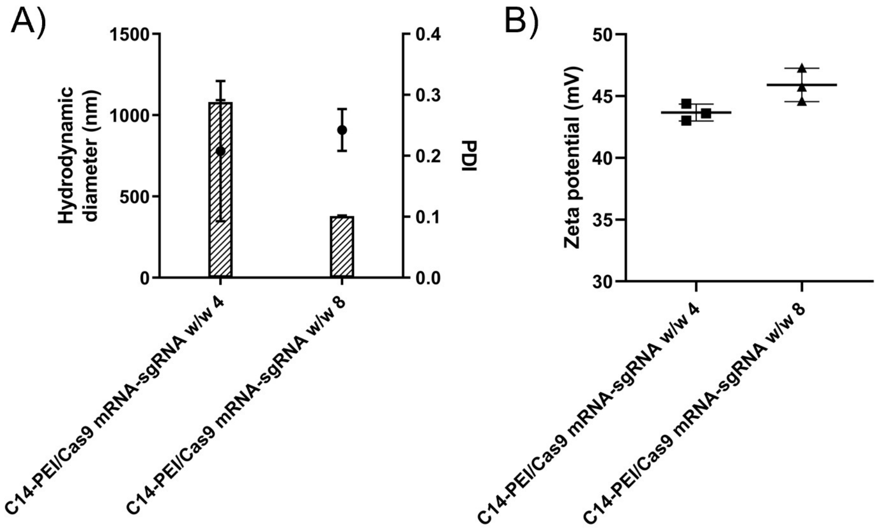

Figure S4. Characterization of C14-PEI micelleplexes co-loaded with Cas9 mRNA and sgRNA. A) the hydrodynamic diameter (bars) and polydispersity index (PDI, dots) of micelleplexes (n=3); **B)** the zeta potential of micelleplexes (n=3).
